# The gut microbiota-bile acid-FXR axis in NAFLD: from progression to therapeutic applications

**DOI:** 10.3389/fphys.2026.1811118

**Published:** 2026-07-08

**Authors:** Wei Zheng, Qian Feng, Juan Wang, Xinpeng Li, Shuo Yin

**Affiliations:** College of Pharmacy, Heze University, Heze, China

**Keywords:** bile acid, farnesoid X receptor, gut microbiota, mechanism, non-alcoholic fatty liver disease

## Abstract

Non-alcoholic fatty liver disease (NAFLD), now recognized as metabolic dysfunction-associated steatotic liver disease (MASLD), has emerged as the predominant chronic liver condition globally. Although pharmacological options have recently emerged for selected patients with MASH and moderate-to-advanced fibrosis, pharmacological treatment remains limited. Recent studies have provided compelling evidence demonstrating that both the gut microbiota and bile acids (BAs) undergo remarkable alterations in NAFLD and contribute substantially to disease progression. The farnesoid X receptor (FXR), a crucial nuclear receptor, plays a central role in the synthesis and metabolism of BAs and also regulates glucose and lipid metabolism while attenuating inflammatory responses. Because of these diverse functions, FXR has become a key focus as a potential therapeutic target for NAFLD. This review provides a comprehensive summary of the interactions between the gut microbiota and BAs, detailing their specific metabolic alterations in NAFLD. It also explains the molecular mechanisms through which FXR regulates glucose and lipid metabolism and offers an up-to-date overview of emerging therapeutic strategies that target the gut microbiota–BA–FXR axis for NAFLD. This review integrates current evidence to clarify how the gut microbiota–BA–FXR axis contributes to NAFLD/MASLD pathogenesis and therapeutic development, which are expected to offer new insights for filling the current unmet clinical need in NAFLD treatment.

## 1 Introduction

Non-alcoholic fatty liver disease (NAFLD) is a highly prevalent chronic liver disease worldwide, imposing an increasingly heavy burden on global public health (Younossi et al., 2023). Without timely and effective clinical intervention, NAFLD can progress along a pathological spectrum from simple hepatic steatosis to non-alcoholic steatohepatitis (NASH), and further develop into liver fibrosis, cirrhosis, and even hepatocellular carcinoma (Friedman et al., 2018). Although pharmacological options have recently emerged for selected patients with NASH and moderate-to-advanced fibrosis, effective, broadly applicable therapies remain limited. Though NAFLD was renamed metabolic dysfunction-associated steatotic liver disease (MASLD) in 2023, the term NAFLD remains widely used in current research and clinical practice (Rinella et al., 2024).

The gut-liver axis, a critical regulatory network mediating bidirectional crosstalk between the intestinal tract and the liver, has become a major focus of research in NAFLD pathogenesis, with the gut microbiota serving as its core functional component (Chen and Vitetta, 2020). The gut microbiota, a highly complex microbial community colonizing the host’s intestinal tract, undergoes profound alterations in NAFLD and plays a pivotal role in its progression. Studies have reported reduced microbial diversity and altered community composition in patients with NAFLD (David et al., 2014). This gut dysbiosis not only impairs intestinal barrier integrity and disrupts systemic immune homeostasis but also modulates the production and metabolic profiling of microbial-derived metabolites, among which bile acids (BAs) play a pivotal role in linking intestinal microbial disorders to hepatic pathological changes (Aron-Wisnewsky et al., 2020; Chen and Vitetta, 2020). BAs are no longer merely regarded as classic mediators of lipid emulsification and absorption; as important endogenous signaling molecules, they also exert crucial regulatory effects on hepatic lipid and glucose metabolism, as well as inflammatory responses (Aron-Wisnewsky et al., 2020; Chen and Vitetta, 2020). Circulating BA profiles exhibit distinct and characteristic alterations in NAFLD patients, with the composition and concentration of BAs closely correlated with disease severity, and specific BA subtypes showing significant associations with key histological features of NAFLD, including lobular inflammation and hepatocyte ballooning (Puri et al., 2018; Gottlieb and Canbay, 2019; Jung et al., 2021; Nimer et al., 2021). Accumulating experimental and clinical evidence has confirmed that modulating the composition of the gut microbiota and restoring BA metabolic homeostasis can effectively intervene in the progression of NAFLD, highlighting the gut microbiota-BA axis as a promising therapeutic target for this disease (Chen and Vitetta, 2020; Chen et al., 2024a).

The farnesoid X receptor (FXR), a nuclear receptor highly expressed in the liver and intestine, is a master regulator of BA homeostasis, mediating the precise feedback regulation of BA *de novo* synthesis and enterohepatic circulation (Chiang, 2013; Chiang and Ferrell, 2018). Beyond its core regulatory role in BA metabolism, FXR also exerts multifaceted and critical effects on systemic glucose and lipid metabolism (Chiang, 2013; Chiang and Ferrell, 2018) and attenuates hepatic inflammatory responses by regulating immune cell activity and the secretion of pro-inflammatory cytokines (Cai et al., 2022). Due to its essential role in multiple pathological processes of NAFLD, FXR has emerged as a core therapeutic target for NAFLD, and targeting FXR to regulate BA metabolism and metabolic homeostasis has shown promising therapeutic potential (Long et al., 2024). Notably, the gut microbiota, BA, and FXR do not act as independent regulatory components in the pathogenesis of NAFLD; instead, they form a tightly interconnected and mutually regulated gut microbiota-BA-FXR regulatory axis (Cai et al., 2022; Collins et al., 2023). In this axis, gut microbial dysbiosis modulates BA metabolic composition and profiling to regulate FXR activation, while FXR signaling in turn shapes the composition and function of the gut microbiota by regulating BA synthesis and excretion, forming a bidirectional crosstalk that drives the initiation and progression of NAFLD (Collins et al., 2023).

This review systematically synthesizes current knowledge on the gut microbiota-BA-FXR axis in NAFLD pathogenesis and elaborates its regulatory mechanisms in detail. It also highlights the latest advances in therapeutic strategies targeting this axis. The purpose of this review is to provide a comprehensive theoretical basis for understanding the molecular mechanisms of NAFLD, and to offer novel insights for developing targeted therapeutic strategies to fill the current clinical gap of lacking effective treatments for this disease.

## 2 BA synthesis, metabolism, and interactions with the gut microbiota

### 2.1 The synthesis and enterohepatic circulation of BAs

**Figure 1:**
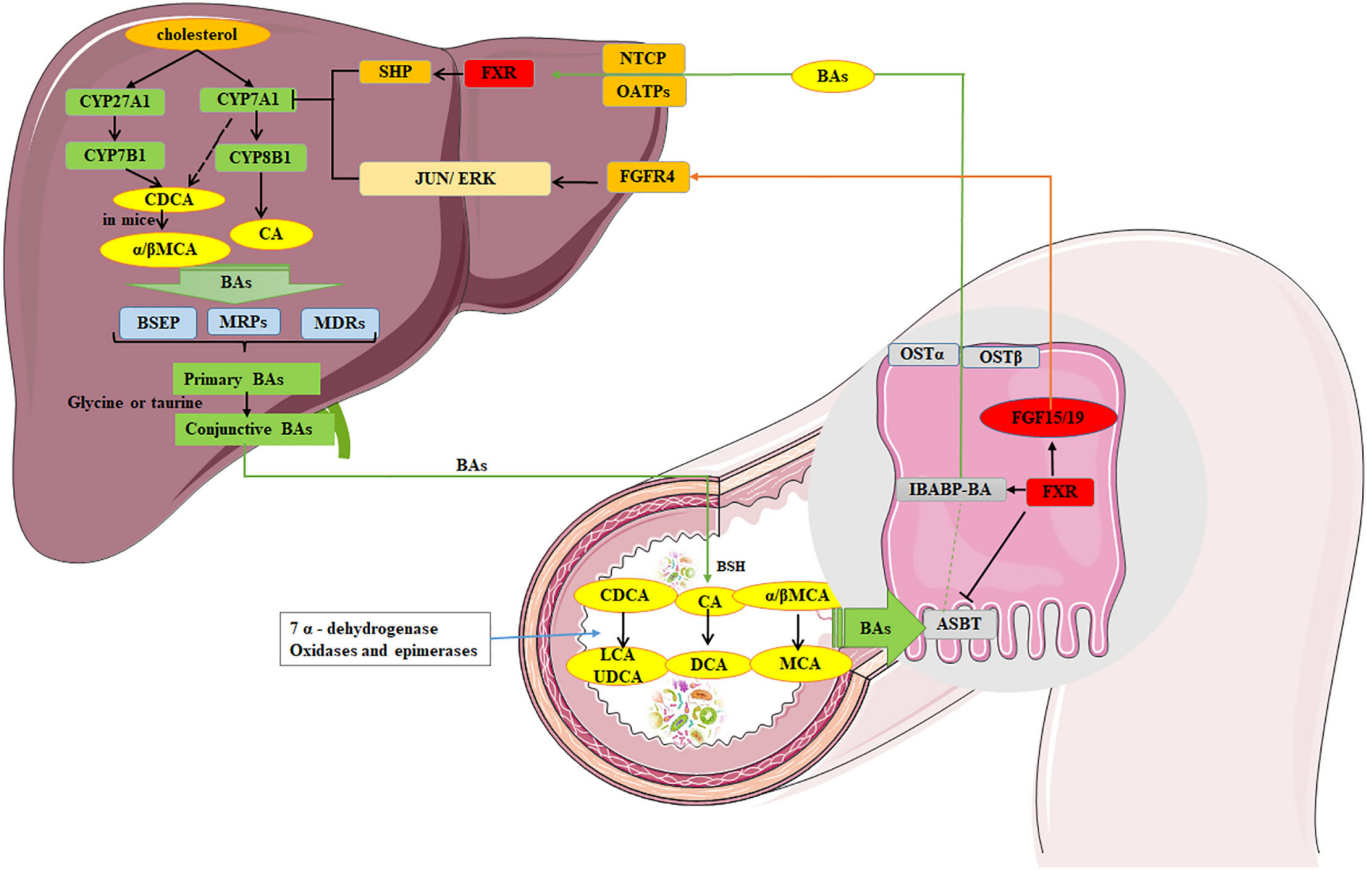
The metabolism and enterohepatic circulation of BAs.

As shown in **Figure 1**, there are two pathways to generate structurally distinct BAs from cholesterol: the classical (neutral) and alternative (acidic) pathways (Chiang and Ferrell, 2018). In the classical pathway, cholesterol is converted into chenodeoxycholic acid (CDCA) and cholic acid (CA) by cholesterol 7α-hydroxylase (CYP7A1) and sterol 12α-hydroxylase (CYP8B1) (Chiang and Ferrell, 2019). CYP7A1 determines the production of total BAs, while CYP8B1 governs the production of CA, a 12α-hydroxylated BA (12-OH BA), and the ratio of CA/CDCA (Chiang and Ferrell, 2018). More than 75% of hepatic BA output is generated through the classical pathway. In the alternative pathway, cholesterol undergoes C27 hydroxylation and 7-hydroxylation catalyzed by sterol 27-hydroxylase (CYP27A1) and oxidized sterol 7α-hydroxylase (CYP7B1), respectively, and ultimately produces CDCA, a non-12-hydroxyl acid (Chiang and Ferrell, 2019). In rodents, CDCA is further converted into muricholic acids (MCAs) (Takahashi et al., 2016). Following synthesis, primary BAs are conjugated with glycine or taurine to increase their hydrophilicity, then transported into bile via the bile acid salt export pump (BSEP) and multidrug resistance protein 2 (MRP2). Upon food intake, BAs are released into the duodenum (Cai et al., 2022). Approximately 95% of BAs are reabsorbed from the intestine via apical sodium-dependent acid transporter (ASBT) and recycled to the liver, and the rest are excreted into feces and urine (Chiang, 2013; Wahlström et al., 2016).

### 2.2 Metabolism of BAs by the gut microbiota

Studies have demonstrated that gut microbiota colonizing the intestinal tract mediate the biotransformation of BAs in the gut (shown in **Table 1**), whereby microbial enzymes deconjugate, dehydroxylate, oxidize, epimerize, and reconjugate BAs (Ridlon et al., 2016). These processes collectively enhance the diversity and hydrophobicity of BAs (Ridlon et al., 2016).

**Table 1:** Metabolism of BAs by the Gut Microbiota.

Modulation	Microbe	BAs	Reference
hydrolysis (deconjugation)	*Bacteroidetes*, *Firmicutes, Actinobacteria*	Tauro/Glyco-BA → UBA:	(Song et al., 2019; Guzior and Quinn, 2021)
7-dehydroxylation	*Clostridium, Eubacterium*	DCA → LCA CA → DCA α-/β-MCA → MDCA	(Ridlon et al., 2006, 2016)
oxidation/epimerization (7-α/β isomerization)	*Clostridium*, *Bacteroides, Ruminococcus*	CDCA → UDCA α-/β-MCA → ω-MCA keto(oxy-)BAs, iso/allo BAs	(Ridlon et al., 2020; Guzior and Quinn, 2021; Liu et al., 2024b)
Desulfation	*Clostridium, Peptococcus, Fusobacterium*, *Proteobacterium*	3-sulfated BA → UBA:	(Quinn et al., 2020; Collins et al., 2023)
Esterification	*Bacteroides*	UBA: → 3-O-acyl ester BA	(Zheng et al., 2024)
Amidation	*Enterocloster*, *Bifidobacterium*, *Bacteroides*, *Enterococcus spp*	UBA → amidated BA	(Quinn et al., 2020; Collins et al., 2023; Rimal et al., 2024)

UBA, unconjugated bile acid.

First, conjugated BAs are hydrolyzed into free forms by bile salt hydrolase (BSH), produced by all major gut microbiota phyla (e.g., *Bacteroidetes*, *Firmicutes*, and *Actinobacteria*) (Song et al., 2019; Guzior and Quinn, 2021). Subsequently, 7α-dehydroxylation, mediated mainly by selected *Clostridium* and *Eubacterium*, mediates the conversion of primary BAs to secondary BAs: specifically, CA to deoxycholic acid (DCA), CDCA to lithocholic acid (LCA) (Collins et al., 2023), and α-/β-MCA to murideoxycholic acid (MDCA) (Ridlon et al., 2006, 2016).

Furthermore, hydroxysteroid dehydrogenases (HSDs), mainly produced by *Clostridium*, *Bacteroides*, and *Ruminococcus*, can reversibly oxidize and epimerize the hydroxyl groups at the C3, C7, and C12 positions of BAs (Ridlon et al., 2020; Guzior and Quinn, 2021). This process leads to the formation of oxy-, iso-, allo-BA derivatives (Liu et al., 2024b), thus may reduce BA hydrophobicity and cytotoxicity (Fuchs et al., 2025). For instance, CDCA can be converted into ursodeoxycholic acid (UDCA) (Guzior and Quinn, 2021). In addition, BA sulfatase, derived from the genera *Clostridium*, *Pneumococcus*, *Fusobacterium*, and *Pseudomonas*, converts BA sulfates into less polar and more easily absorbable desulfated BA (Gérard, 2013).

Recent studies have further demonstrated that unconjugated BAs undergo reconjugation with fatty acids or amino acids in the intestine, with the involvement of gut microbiota (Zheng et al., 2024). Specifically, CA, CDCA, DCA, LCA, isoDCA, and isoLCA conjugate with short-chain fatty acids (SCFAs) and long-chain fatty acids (LCFAs, mainly C16:0 and C18:0) via esterification, a process catalyzed by *Bacteroides* (Zheng et al., 2024). Additionally, *Bifidobacterium*, *Bacteroides*, and *Enterococcus* spp catalyze unconjugated BAs reconjugation with amino acids at the C24 acyl position into additional amino acid-conjugated bile acids (AABAs) (Quinn et al., 2020; Collins et al., 2023; Rimal et al., 2024).

### 2.3 Regulation of the gut microbiota by BAs

BAs exhibit bidirectional effects on regulating the gut microbiota by shaping its diversity, abundance, and metabolic functions (Collins et al., 2023). First, hydrophobic BAs exert selective antimicrobial effects by disrupting bacterial cell membrane integrity (Coleman et al., 1980; Heuman et al., 1996), thereby triggering cytoplasmic leakage, DNA damage, protein misfolding, and oxidative stress (Fujisawa and Mori, 1997). Additionally, BAs can activate FXR to induce antimicrobial peptides and lectin production, thereby indirectly limiting bacterial overgrowth and maintaining intestinal barrier homeostasis (Inagaki et al., 2006). A recent study demonstrated that DCA, CDCA, and LCA selectively inhibit specific strains of *Firmicutes* and *Bacteroidetes* while simultaneously promoting the proliferation of *Proteobacteria* and *Akkermansia muciniphila*, with DCA exerting the greatest impact (Peng et al., 2024). In contrast, UDCA and TUDCA promote the growth of *Bifidobacterium pseudonumeratum* and *Bifidobacterium pseudopodium* (Peng et al., 2024). However, more in-depth research is warranted to elucidate the impacts of BA on specific gut microbiota and the underlying mechanisms.

## 3 Regulation of BAs on lipids and glucose metabolism

As major bioactive components of bile, BAs not only participate in lipid emulsification and absorption (Gottlieb and Canbay, 2019) but also function as signaling molecules by interacting with specific receptors, especially FXR and Takeda G protein-coupled receptor 5 (TGR5) (Chiang and Ferrell, 2018). Through these interactions, BAs play a crucial role in regulating their own biosynthesis, modulating lipid metabolism and glucose homeostasis, and influencing inflammatory responses (Rajani and Jia, 2018; Cai et al., 2022).

### 3.1 Regulation of lipid metabolism by FXR

**Figure 2:**
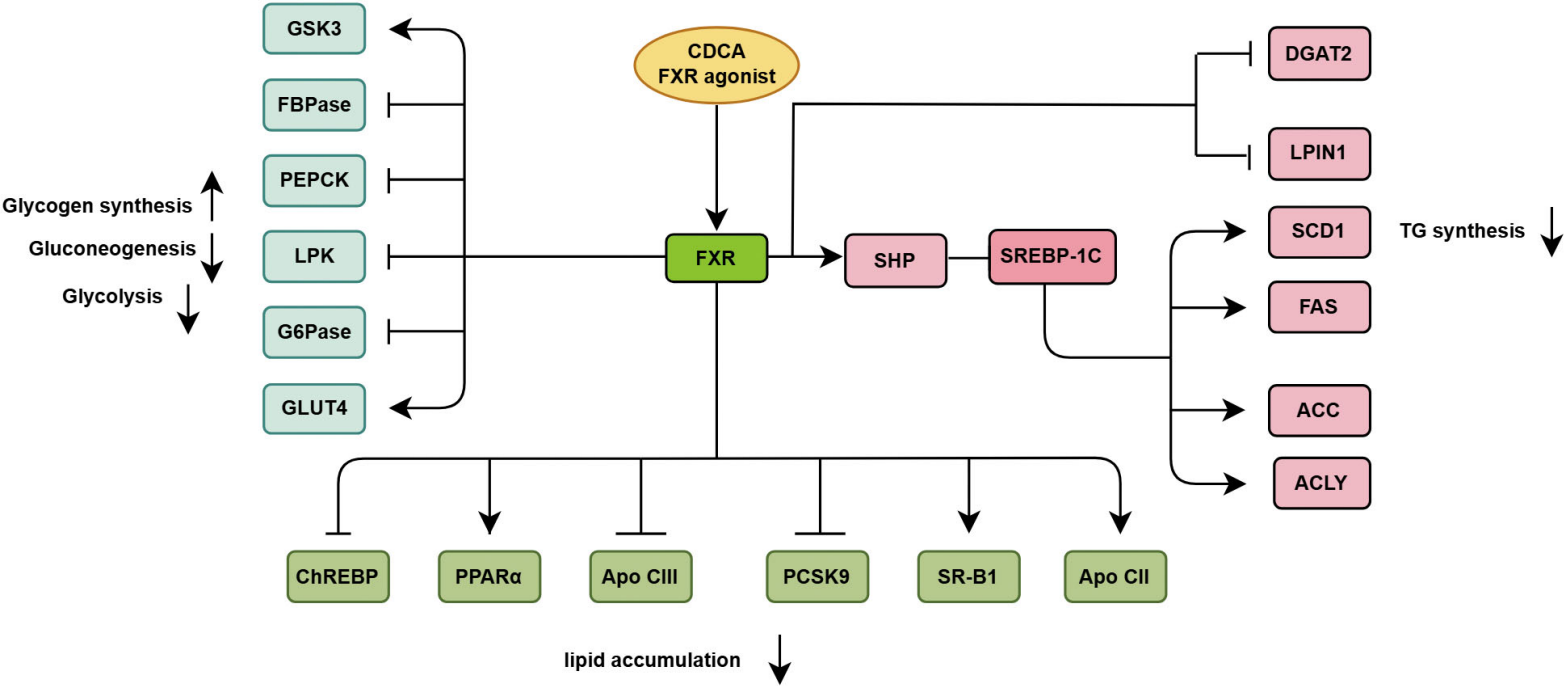
Regulations of BAs on lipids and glucose metabolism via FXR.

Accumulating studies have demonstrated that FXR plays a crucial role in cholesterol catabolism, transport, lipogenesis, and triglyceride (TG) metabolism (de Aguiar Vallim et al., 2013; Katafuchi and Makishima, 2022). As depicted in **Figure 2**, these regulatory effects are primarily mediated through multiple molecular pathways:

(1) Activation of hepatic FXR induces the expression of small heterodimer partner (SHP), which inhibits the transcription of sterol regulatory element-binding protein 1c (*SREBP-1c*) (Watanabe et al., 2004). Meanwhile, activation of the fibroblast growth factor (FGF) 15/19–FGFR4/β-Klotho–ERK pathway also represses SREBP-1c activity (Fleishman and Kumar, 2024). Collectively, these repressions downregulate the transcription of genes encoding lipogenic enzymes such as fatty acid synthase (*FAS*), acetyl-CoA carboxylase (*ACC*), stearoyl-CoA desaturase 1 (*SCD1*), and ATP citrate lyase (*ACLY*), thereby suppressing hepatic TG synthesis and attenuating lipid accumulation in hepatocytes (Watanabe et al., 2004; Fleishman and Kumar, 2024; Li et al., 2024). Furthermore, activated FXR upregulates peroxisome proliferator-activated receptor alpha (PPARα), thereby increasing the expression of rate-limiting enzymes involved in the fatty acid β-oxidation pathway. This process not only suppresses *de novo* lipogenesis but also enhances fatty acid catabolism, ultimately contributing to reduced hepatic lipid accumulation (Cheng and Klaassen, 2008).

(2) Activation of the FXR signaling pathway induces the expression of apolipoprotein C-II (ApoC-II), while simultaneously suppressing the expression of apolipoprotein C-III (ApoC-III). This dual regulatory effect enhances the activity of lipoprotein lipase (LPL), thereby promoting the hydrolysis and clearance of TG. Consequently, this mechanism reduces TG accumulation (Ghosh Laskar et al., 2017; Sun et al., 2021).

(3) FXR activation not only induces the expression of scavenger receptor class B type 1 (SR-B1) to facilitate hepatic uptake of high-density lipoprotein (HDL) from the systemic circulation, but also regulates genes related to cholesterol and lipoprotein metabolism, including the very low-density lipoprotein receptor (VLDLR), proprotein convertase subtilisin/kexin type 9 (PCSK9), to reduce cholesterol levels (Fleishman and Kumar, 2024).

Notably, FXR exerts tissue-specific regulatory effects on lipid metabolism. Both hepatic FXR activation and intestinal FXR inhibition suppress TG synthesis, thereby reducing hepatic TG accumulation. Sinal et al. (2000) reported that liver-specific *Fxr* knockout mice exhibited elevated hepatic lipids, plasma TG, and cholesterol; conversely, mice treated with BAs or FXR agonists showed reduced levels (Zhang et al., 2006; Fiorucci et al., 2009). Huang et al. (2019) observed that inhibiting intestinal FXR increases hepatic BA synthesis and fecal excretion, effectively lowering the plasma and hepatic cholesterol levels. Wu et al. (2021b) found that intestinal FXR deficiency (in *Fxr*^ΔIE^ *ApoE*^–/–^ mice) or direct FXR inhibition (via treatment with the FXR antagonist) reduced ceramide levels by suppressing intestinal FXR-FGF19 signaling and enhancing CYP7A1-mediated cholesterol catabolism.

### 3.2 Regulation of glucose metabolism by FXR

Specifically, BAs regulate glucose homeostasis through two complementary mechanisms: direct activation of FXR and indirect induction of intestinal FGF15/19 (FGF15 in rodents and FGF19 in humans) (Katafuchi and Makishima, 2022). This dual regulatory mechanism enhances glycogen synthesis, modulates hepatic gluconeogenesis and glycolytic gene expression, and improves insulin sensitivity, thereby counteracting insulin resistance and stabilizing circulating glucose levels (Fiorucci and Distrutti, 2022; Wei et al., 2024). Consistent with its regulatory pattern in lipid metabolism, activated FXR downregulates key gluconeogenic enzymes, including glucose-6-phosphatase (G6Pase), fructose-1,6-bisphosphatase (FBPase), and phosphoenolpyruvate carboxykinase (PEPCK), which collectively reduce hepatic glucose production (Fleishman and Kumar, 2024). Furthermore, FXR activation decreases the expression of Liver-type pyruvate kinase (L-PK), a critical gene involved in the glycolysis pathway, which inhibits glycolysis and promotes glycogen storage (Duran-Sandoval et al., 2005). In hepatocytes, FXR induces the expression of SHP, which interacts with HNF4α and forkhead box protein O1 (FOXO1) to repress the promoter activity of PEPCK and fructose 1,6-bisphosphatase, further curbing gluconeogenesis (Yamagata et al., 2004; Wattanavanitchakorn et al., 2018; Zhang et al., 2018).

Hepatic FXR activation or overexpression leads to reduced circulating glucose levels and improved insulin sensitivity; *Fxr*^−/−^ mice exhibit glucose intolerance and insulin resistance, whereas treatment with the FXR agonist GW4064 ameliorates insulin resistance and restores glucose homeostasis (Cariou et al., 2006). In contrast, intestinal FXR inhibition enhances glucagon-like peptide-1 (GLP-1) secretion and insulin release, thereby alleviating insulin resistance and glucose dysregulation in diabetic murine models (Yan et al., 2022). Specifically, inhibition of intestinal FXR signaling by glycine-β-muricholic acid (G-β-MCA) prevents high-fat diet (HFD)-induced obesity, insulin resistance, and hepatic steatosis in mice (Jiang et al., 2015), further confirming the tissue-specific regulatory role of FXR in metabolic homeostasis.

**Figure 3:**
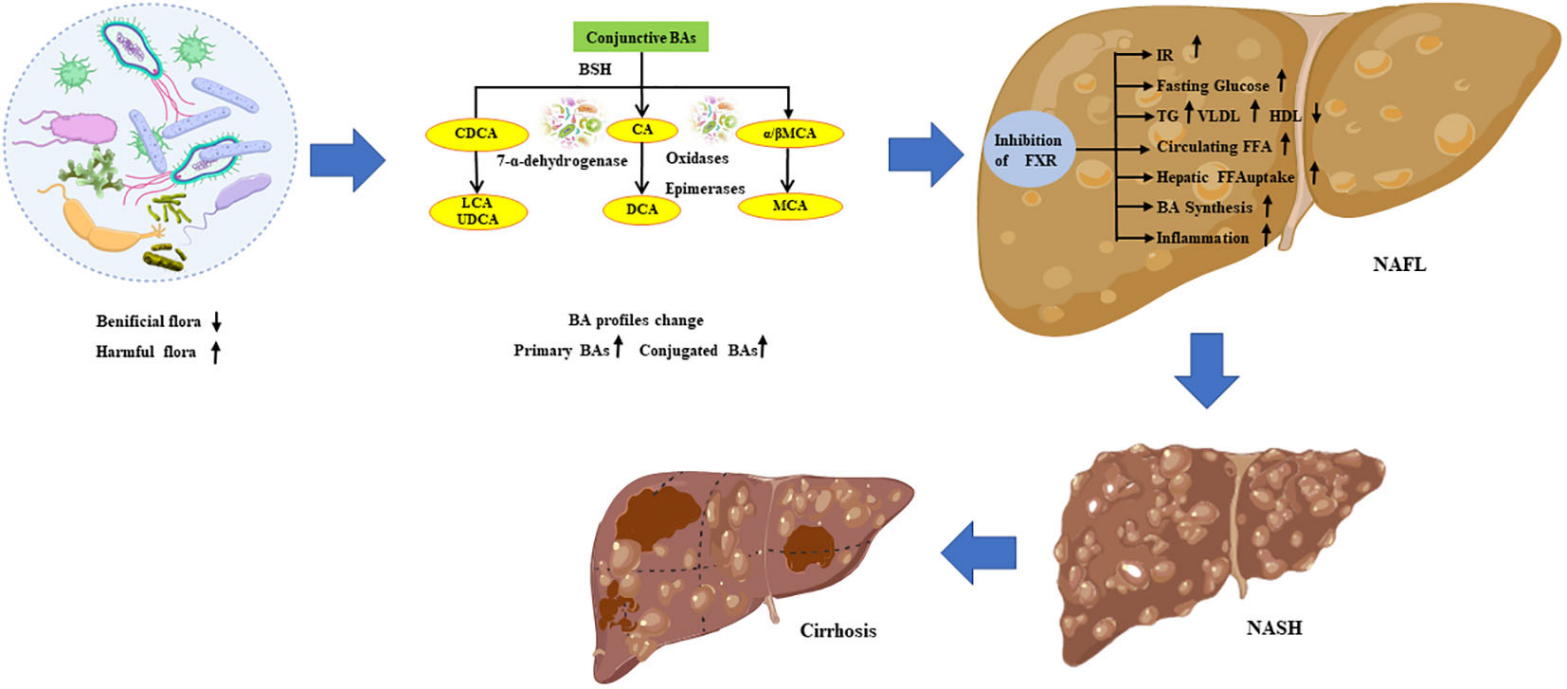
The role of gut microbiota-BAs-FXR axis in NAFLD progression.

Building on these findings regarding FXR-mediated metabolic regulation, FXR agonists and FGF15/19 analogs exhibit considerable therapeutic potential for the treatment of metabolic syndrome and NAFLD (Katafuchi and Makishima, 2022). Specifically, activation of intestinal FXR stimulates the production of FGF15 in obese mice, which reduces systemic ceramide levels and hepatic glucose output, improves glucose homeostasis and insulin resistance, and increases energy expenditure in adipose tissue, collectively improving systemic glucose and lipid homeostasis (Fang et al., 2015; van Zutphen et al., 2019). Zhao et al. (2021) found that both FGF15 overexpression and its administration significantly reduce fasting glucose levels, insulin resistance, TG, and free fatty acid levels in HFD mice. Furthermore, supplementary FGF19 could physiologically repress hepatic lipogenesis in NAFLD mice by activating SHP and DNA methyltransferase-3a, which are likely dysregulated in NAFLD (Kim et al., 2020); central administration of FGF19 suppresses hepatic gluconeogenesis and promotes lipolysis in type 1 diabetes (T1D) rats (Zhang and Yang, 2024). In mice, activation of intestinal FXR stimulates FGF15, whereas in humans, FGF19 mediates these effects; thus, findings in rodent models may not directly translate to human physiology. The role of the gut microbiota–BAs–FXR axis in NAFLD progression is illustrated in **Figure 3**.

## 4 Alterations of BA profiles and gut microbiota in NAFLD

### 4.1 Alterations in BA Profiles in NAFLD

Patients with NAFLD show increased BA levels and alterations in BA profiles (Nimer et al., 2021), which are closely linked to hepatic lipid accumulation, inflammation, and fibrosis, making BA profiles a potential biomarker of NAFLD progression (Browning et al., 2019).

Studies have uncovered increased BA synthesis in NAFLD: both serum and liver BA levels are significantly elevated. Specifically, the classical pathway is abnormally hyperactive, while the alternative pathway is relatively reduced. Notably, patients with NASH exhibit 9.3-fold increased expression of CYP7A1 in the liver, along with 2.83-fold increased expression of CYP27A1 and 2.18-fold increased expression of CYP8B1, which leads to increased levels of DCA and decreased levels of CDCA in serum (Jiao et al., 2018). In HFD-induced rats and Western diet-induced mice, the levels of 12-OH BAs, such as CA and DCA, are significantly elevated (Nimer et al., 2021), while the levels of non-12-OH BAs, such as CDCA and UDCA, are relatively reduced (Chevre et al., 2018; Gillard et al., 2022), leading to an elevated ratio of 12-OH BAs/non-12-OH BAs. However, this elevated ratio is only observed in males with dyslipidemia (Liu et al., 2025) or type 2 diabetic (T2D) patients with insulin resistance (Haeusler et al., 2013). Moreover, in NAFLD patients, free fatty acids (FFAs) can abrogate the inhibitory effect of SHP on CYP7A1 and sodium taurocholate cotransporting polypeptide (NTCP) (Bechmann et al., 2013; Fang et al., 2024), further promoting BA synthesis and hepatocellular uptake.

Previous studies revealed an impaired BA transport in NAFLD: the expression of NTCP is increased, whereas BSEP and MRP2 is decreased (Puri et al., 2018; Liu et al., 2023; Murphy et al., 2024). This imbalance results in intrahepatic BA accumulation and the development of microcholestasis (Liu et al., 2023), especially the accumulation of 12-OH BAs such as CA and DCA, which contribute to hepatocyte apoptosis, hepatic inflammation, and fibrosis (Bertaggia et al., 2017).

Because of the impaired activity of BSH and 7α-hydroxysteroid dehydrogenase resulting from the gut dysbiosis (Boursier et al., 2016; Collins et al., 2023), the ratios of primary to secondary BAs and conjugated to unconjugated BAs are significantly altered in NAFLD (Yu et al., 2021; Basuni et al., 2024), which is closely associated with the presence and severity of NASH and fibrosis (Puri et al., 2018; Smirnova et al., 2022).

### 4.2 Alterations of gut microbiota in NAFLD

Compared to healthy individuals, patients with NAFLD exhibit a marked reduction in gut microbiota diversity and distinct alterations in community composition (Xu et al., 2024). Some studies have reported that the abundance of *Firmicutes* increased and *Bacteroidetes* decreased in the NAFLD context, and the ratio of *Firmicutes*/*Bacteroidetes* elevated (Hoyles et al., 2018), which is closely correlated with the severity of hepatic steatosis (Jasirwan et al., 2021). Nevertheless, conflicting results have also been documented (Vallianou et al., 2021).

Research indicates that individuals in industrialized countries exhibit a higher *Bacteroidetes*/*Firmicutes* ratio (Gomaa, 2020). Dietary patterns significantly modulate this ratio: Western diets are associated with an elevated *Bacteroidetes*/*Firmicutes* ratio, whereas plant-based and Mediterranean dietary patterns tend to reduce it (Soldán et al., 2024). Environmental factors also play a role—anaerobic bacteria predominate at high altitudes, while cold climates favor the expansion of *Firmicutes* (Soldán et al., 2024). Furthermore, aging exerts a marked influence on gut microbial ecology: compared with younger individuals, the elderly population(aged ≥65 years) displays reduced gut microbial diversity and a compositional shift toward facultative anaerobes (Salazar et al., 2017).

Recent studies have identified a close association between gut microbial dysbiosis and the pathological progression of NAFLD. During the early stage of NAFLD, microbiota profiling shows a significant elevation in *Prevotella*, *Bacteroides*, and *Streptococcus*, accompanied by reduced representation of *Lactobacillus*, *Ruminococcus*, *Clostridium*, and *Akkermansia muciniphila* (Wu et al., 2021a). This dysbiotic pattern becomes more pronounced in NASH, characterized by increased levels of the phyla *Firmicutes* and *Proteobacteria* (Aron-Wisnewsky et al., 2020) and a marked depletion of *Faecalibacterium prausnitzii*, *Akkermansia muciniphila*, *Bacteroides uniformis*, and *Bifidobacterium bifidum* (Xu et al., 2024). In patients with NAFLD-related cirrhosis, the abundances of *Lachnospiraceae*, *Ruminococcaceae*, and *Clostridium* clusters XIV and IV are relatively lower (Aron-Wisnewsky et al., 2020); However, the abundance of *Bacteroidaceae*, *Enterobacteriaceae*, and *Streptococcus* is elevated (Ponziani et al., 2019).

Notably, emerging evidence highlights that gut dysbiosis drives perturbations in key microbial metabolites, such as lipopolysaccharides (LPS), BAs, short-chain fatty acids (SCFAs), trimethylamine oxide (TMAO), and ethanol (Vallianou et al., 2021). These metabolic alterations induce intestinal barrier dysfunction, translocation of intestinal bacteria, and immune dysregulation through multiple interconnected pathways (Ji et al., 2019), collectively exacerbating hepatic injury, inflammatory responses, and fibrogenesis (Aron-Wisnewsky et al., 2020; Chen and Vitetta, 2020).

## 5 Targeting gut microbiota and BA metabolism for NAFLD therapy

Lifestyle modification, including dietary intervention and regular physical activity, remains the cornerstone of NAFLD management. However, this lifestyle modification is not easy to maintain. Unfortunately, approved pharmacological options remain limited to specific patient populations. Therapeutic agents or probiotics that modulate FXR activation, restore gut microbiota homeostasis, and rebalance BA metabolism are recognized as promising strategies to treat NAFLD (Sun et al., 2021; Carpi et al., 2022; Fogelson et al., 2023; Perler et al., 2023; Wei et al., 2024).

As shown in **Table 2**, a series of FXR agonists are currently under clinical investigations for NAFLD and show potential therapeutic effects, such as EDP-305 (Ratziu et al., 2022), tropifexor (Sanyal et al., 2023), cilofexor (Alkhouri et al., 2022), INT-767 (Wang et al., 2022), fexaramine, and OCA, and BA transporter-targeting agents-volixibat (Newsome et al., 2020). Despite evidence of fibrosis improvement in some analyses, OCA—a representative FXR agonist—failed to obtain FDA approval for NASH due to an overall benefit–risk profile deemed insufficient (Rinella et al., 2022). Notably, the intestine-specific FXR agonist fexaramine has been demonstrated to increase the abundance of *Acetatifactor* spp. and *Bacteroides* that produce LCA, elevate serum UDCA, taurolithocholic acid (TLCA), and colonic DCA (Pathak et al., 2018). Additionally, fexaramine promotes FGF15 and GLP-1 secretion in obese and diabetic mice, thereby improving lipid levels, glucose tolerance, and insulin sensitivity (Pathak et al., 2018).

**Table 2:** Summary of FXR agonists in clinical development for NAFLD/NASH.

FXR agonist	Mechanism of action	Trial Phase & design	Efficacy outcomes	Adverse events	Reference
Obeticholic acid (OCA)	Steroidal FXR agonist; activates FXR in liver and intestine, suppresses CYP7A1, reduces BA synthesis, and inhibits SREBP-1c to decrease lipogenesis	Phase 3 (REGENERATE: multicenter, randomized, double-blind, placebo-controlled)	• Fibrosis improvement ≥1 stage without NASH worsening: 22.4% (OCA 25 mg) vs 9.6% (placebo), P<0.0001 • NASH resolution without fibrosis worsening: 6.5% vs 3.5%, P=0.093 • Antifibrotic effect and long-term tolerability • supported by >8,000 patient-years of exposure	Pruritus (most common); dyslipidemia; cholelithiasis; hepatotoxicity risk; global FXR activation may disrupt cholesterol homeostasis; no significant differences in TEAEs, serious TEAEs, or mortality vs placebo	(Fiorucci et al., 2025)
Tropifexor (LJN452)	Non-steroidal potent FXR agonist; activates FXR/FGF15/19 signaling, suppresses hepatic BA synthesis, reduces liver fat content and transaminases	Phase 2a/b (FLIGHT-FXR: adaptive, randomized, double-blind, placebo-controlled; NCT02855164)	• ALT reduction: ~15 U/L greater than placebo at higher doses • Hepatic fat fraction (HFF) reduction: –19.07% (140 µg), –39.41% (200 µg) vs –10.77% • (placebo); improvements sustained to week 48 • Dose-dependent reduction in ALT and liver fat	Pruritus (most common, dose-dependent; higher frequency at higher doses) Combination with licogliflozin (NCT04065841) terminated	(Yoneda et al., 2023; Li et al., 2026)
Cilofexor (GS-9674)	Non-steroidal selective FXR agonist; targets hepatic and intestinal FXR, modulates BA metabolism and lipid homeostasis; synergistic with ACC inhibitors	Phase 2 (open-label, proof-of-concept; NCT03987074)	• Triple therapy (semaglutide + cilofexor 30 or 100 mg + firsocostat) improved liver fat (MRI-PDFF change: –9.8 to –11.0% vs –8.0% with semaglutide alone), liver biochemistries, and noninvasive fibrosis markers • Combination with fenofibrate effectively mitigated hypertriglyceridemia induced by ACC inhibitor (Phase 2 RCT, NCT02781584)	Treatment-related AEs in 73–90% (mostly gastrointestinal: nausea, diarrhea) Generally well tolerated; no ethnic differences in Japanese vs non-Japanese subjects	(Patel et al., 2020; Li et al., 2026)
EDP-305	Oral non-steroidal FXR agonist; modulates BA and lipid metabolism via FXR activation, ameliorates hepatic inflammation and fibrosis	Phase 2 (randomized, double-blind, placebo-controlled, dose-ranging; NCT03421431)	• ALT reduction: –27.9 U/L (2.5 mg) vs –21.7 U/L (1 mg) vs –15.4 U/L (placebo); P=0.049 for 2.5 mg vs placebo • Absolute LFC reduction: –7.1% (2.5 mg, P=0.0009) vs –3.3% (1 mg) vs –2.4% (placebo) • Supports further long-term histologic endpoint trials	Pruritus (most common): 50.9% (2.5 mg → discontinuation 20.8%), 9.1% (1 mg), 4.2% (placebo); also nausea, vomiting, diarrhea, headache, dizziness	(Ratziu et al., 2022)
INT-787	Novel hydrophilic semi-synthetic BA derivative; FXR agonist (also dual FXR/TGR5 agonist in preclinical models); reduces inflammation and fibrosis markers, modulates BA/lipid metabolism	Phase 1 (first-in-human, randomized, placebo-controlled, single/multiple ascending doses)	• Well tolerated after single and multiple oral doses in healthy volunteers • Target engagement confirmed by decreased C4 and increased FGF-19 levels • Steady-state INT-787 reached by day 7; half-life 21–55 h at doses >50 mg; Cmax ~2-fold higher under fasting vs fed conditions	Only 4 mild, transient pruritus events reported at higher doses (no intervention needed) Generally well tolerated; dual FXR/TGR5 agonism may improve metabolic profile	(Capozza et al., 2025)
INT-767	Dual FXR/TGR5 agonist; activates FXR (BA/lipid metabolism) and TGR5 (energy expenditure, browning, insulin sensitivity)	Phase 1 (Phase 1 completed; Phase 2 for NASH not yet fully conducted)	In high-fat diet-induced NASH rats: INT-767 significantly alleviates high-fat caused liver damage characterized with lipid accumulation and hepatic infiltration of immune cells, and robustly restores the lipid, glucose metabolism to normal level, attenuates insulin resistance.	Not available	(Rizzo et al., 2010; Hu et al., 2018)
MET409 (Omesdafexor)	Structurally optimized non-bile-acid FXR agonist	Phase II proof-of-concept trial; 80 mg/50 mg/placebo; 12 weeks	Relative liver-fat reductions: 55 % (80 mg) and 38 % (50 mg) vs 6 % placebo; ≥30 % reduction achieved in 93 % and 75 % vs 11 %	Mild-to-moderate pruritus: 16 % (50 mg) and 40 % (80 mg); LDL-C increased and HDL-C decreased	(Harrison et al., 2021)
Nidufexor (LMB763)	Partial FXR agonist/modulator	Phase II CLMB763X2201; 100 mg/50 mg vs placebo; 12 weeks	≥30 % liver-fat reduction: 70 % (100 mg) and 50 % (50 mg) vs <5 % placebo; body-weight loss of 1.44 kg and 2.02 kg	Pruritus: 54.1 % (100 mg), 29.5 % (50 mg), 15 % placebo; ~32 % of the high-dose arm required dose reduction or discontinuation	(Chianelli et al., 2020; Li et al., 2026; Shi et al., 2026)
Vonafexor (EYP001)	Second-generation non-steroidal FXR agonist	Phase IIa LIVIFY; 100/200 mg vs placebo; 12 weeks	Absolute MRI-PDFF decrease 6.3 % (100 mg) and 5.4 % (200 mg) vs 2.3 % placebo; ≥30 % liver-fat reduction in 50 % and 39.3 % vs 12.5 %	Pruritus: 9.7 % (100 mg), 18.2 % (200 mg) vs 6.3 % placebo; mostly mild	(Ratziu et al., 2023)
TERN-101	Liver-selective non-bile-acid FXR agonist	Phase IIa LIFT; 5/10/15 mg vs placebo; 12 weeks	Mean cT1 reduction 38–74 ms; relative MRI-PDFF decline 12.9–19.7 % vs 8.4 % placebo; ALT decreased by 13–18 %	Pruritus: 16 % (5 mg), 11.5 % (10 mg), 17.4 % (15 mg) vs 0 % placebo; slight LDL-C rise in the 15 mg arm	(Terns Pharmaceuticals, 2021)

Preclinical studies suggest that traditional Chinese medicine (TCM) formulations and TCM-derived compounds may ameliorate NAFLD-related phenotypes by targeting gut microbiota dysbiosis and BA metabolism (shown in **Table 3**). Zexie-Baizhu Decoction effectively attenuates hepatic injury, reduces lipid accumulation, and downregulates pro-inflammatory cytokine expression in NAFLD models (Cao et al., 2022). Subsequent studies attributed these effects to its ability to promote the growth of *Akkermansia muciniphila* and restore the *Firmicutes*/*Bacteroidetes* ratio (Shi et al., 2025). Shenling Baizhu Powder significantly enriches the relative abundance of beneficial bacteria (*Bifidobacterium* and *Lactobacillus*), suppresses the growth of pro-inflammatory bacteria (*Erysipelatoclostridium* and *Lachnoclostridium*), and improves BA/lipid metabolic profiles, thereby alleviating the symptoms of NAFLD in HFD-induced mice (Chen et al., 2024a). Additionally, Linggui Shu Gan Tang (Chen et al., 2024b) and Tianhuang Fang (Luo et al., 2023) have been shown to mitigate NAFLD progression by modulating gut microbiota dysbiosis and BA metabolism. Astragaloside IV has been shown to ameliorate hepatic steatosis by reducing the abundance of BSH-producing bacteria and increasing T-β-MCA levels in NAFLD mice, thereby suppressing intestinal FXR signaling (Zhai et al., 2022). Similarly, ganoderic acid regulates BA and fatty acids synthesis, alleviates excessive hepatic lipid droplets accumulation, and reshapes gut microbial composition in HFD-induced mice (Guo et al., 2020).

**Table 3:** Therapeutic effects of TCM related to gut microbiota and BAs for NAFLD.

Medicine	Dosage and administration	Models	Effects on NAFLD	Effects on gut microbiota	Effects on bile acids	Reference
Astragaloside	100 µl of AS-IV at 12.5, 25, 50 mg/kg/day by oral gavage for 12 weeks	HFD-diet C57BL/6N male mice	body weight ↓, liver index ↓, eWAT ↓ serum TC↓, TG ↓, ALT↓, AST↓, LDL-C ↓ Liver steatosis ↓, adipocyte size ↓ mRNA expression of SREBP-1c and FAS in the liver ↓ GLP-1 ↑, ceramide ↓	genus of *Lactobacillus*, *Enterococcus*, *Streptococcus*, *Bacteroide*s, *Lactococcus*, *Allobaculum*, *Adlercreutzia* ↓ microbial diversity ↑	the ratio of unconjugated BAs to conjugated BAs in the distal ileum content, the primary-to-secondary BAs ratio ↓ intestinal T-β-MCA, TUDCA, GCDCA ↑, CA and DCA ↓ the activity of FXR in the ileum ↓, FGF15 ↓, the activity of hepatic FXR ↑	(Zhai et al., 2022)
Hyperoside	1.5, 3.0 mg/kg/day by oral gavage for 6 weeks	HFD-diet Wistar male rats	hepatocyte hydropic degeneration and cell swelling ↓, hepatic lipid droplets ↓ serum and hepatic TG ↓, Apo A↑, Apo C ↓, Apo B ↓, Apo E↓, VLDL ↓, hepatic TC↓	*Enterococcus* and *Bacteroides* ↓, *Bacillus* ↓, *Lactobacillus* ↓, *Lactococcus* ↓, *Streptococcus* ↓, *Bifidobacterium*, and *Clostridium*↓	total and unconjugated BAs levels in the liver and serum ↓, fecal conjugated and total BAs levels ↑	(Wang et al., 2026)
Ganoderic acid A	15, 75mg /kg/day by oral gavage for 8 weeks	HFD-diet Kunming male mice	body weight↓, liver index, eWAT, adipocyte diameter↓ serum TC, TG, LDL-C ↓, HDL-C ↑ hepatic TC ↓, TG ↓	restore HFD-disrupted gut microflora. *Eisenbergiella tayi*, *Alistipes senegalensis*, *Oscillibacter valericigenes*, *Marvinbryantia* *formatexigen*, *Bacteroides acidifaciens*, *Mucispirillum schaedleri*, *Bacteroides eggerthii* ↑, 3. *Parabacteroides* *goldesteinii*, *Anaerotruncus colihominis*, *Barnesiella intestiniho-mini*, *Lactobacillus murinus*, *Clostridium clariflavum* ↓.	hepatic BAs ↓ fecal BAs ↑	(Guo et al., 2020)
Schisandra chinensis lignans	20, 40, 80 mg/kg/day by gavage for 4 weeks	MCD diet C57BL/6J male mice	body weight and eWAT ↓ serum ALT↓, AST↓, TC ↑, HDL-C ↑ hepatic lipid droplet accumulation and inflammatory cell infiltration ↓ hepatic TNF-α ↓, IL-1β ↓, CCL2↓ the phosphorylation of key components involved in NF-κB signaling↓, the expression of fatty acid oxidation-related enzymes ↑	alpha diversity ↑ *Firmicutes* ↓, *Bacteroidetes* ↑, Lachnospiraceae_NK4A136 _group and Alistipes ↑,	hepatic BAs ↓, HCA and most of the secondary BAs ↓ fecal cholic acid glucuronide ↓, DCA↑	(Yan et al., 2025)
*Stevia rebaudiana* root polysaccharide	100, 200, 400 mg/kg/day by gavage for 8 weeks	HFD-diet C57BL/6J male mice	body weight, eWAT ↓ serum ALT↓, AST↓, AST/ALT↓, TC↓, TG↓, LDL-C↓, HDL-c ↑ hepatic MDA↓, TNF-α↓, IL-1β↓, IL-16↓, MCP-1↓, ox-LDL↓, SOD↓, GSH-Px ↑ 3. hepatic fat accumulation↓, the diameter and weight of white fat vacuoles↓, fat vacuoles and lipid droplets ↓	F/B ratio ↓, *Desulforibrio*, *Clostridium*, and *Enterobacteriaceae* ↓, *Butyricimonas*, *Parabacteroides*, and *Prevotella* ↑	circulating TCDCA ↓, CA ↑, CDCA↑	(Bao et al., 2025)
chicory extract	100, 200, 400 mg/kg/day by gavage for 8 weeks	HFD-diet C57BL/6J male mice	1. body weight↓, liver weight ↓, bile weight ↑, plasma TC and LDL-C↓ 2. fatty vacuolar degeneration and lipid droplet accumulation ↓, the NAFLD activity score ↓	1. *Bacteroides* ↑, *Lactobacillus* ↑, and *Bifidobacterium* ↑ 2. Actinobacteriota ↑, Desulfobacterota↓, Muribaculaceae ↑, Desulfovibrionaceae ↓,	plasma TDCA ↑	(Wang et al., 2025)
Zexie-Baizhu Decoction	1500 mg/kg/day by gavage for 29 weeks	HFD-diet C57BL/6J male mice	body weight↓, liver weight ↓, eWAT ↓ serum ALT↓, AST↓, TC↓, TG↓, LDL-C↓ fasting blood glucose levels↓, glucose tolerance ↑ 3. hepatic fat accumulation↓, liver steatosis↓, hepatocellular ballooning↓, lobular inflammation↓	F/B ratio ↓, *Akkermansia muciniphila* ↑	total BA levels in feces and plasma↓ the ratio of LCA/(LCA + CDCA) and LCA/(LCA + UDCA) in feces ↓ the ratio of primary BAs to secondary BAs, the ratio of unconjugated to conjugated BAs in plasma ↑	(Shi et al., 2025)
Shenling Baizhu San	21.8 g/kg/day by oral gavage for 6 weeks	Western-diet with a high sugar solution containing 23.1 g/L d-fructose and 18.9 g/L d- glucose, 0.2 µL/g CCl4 intraperitoneal injection every week, C57BL/6J male mice	1. body weight↓, liver weight↓, eWAT ↓, insulin resistance ↑ 2. hepatic TC↓, TG↓, serum ALT↓, AST↓, the mRNA expressions of IL-10↓, IL-6↓, MCP-1↓, IL-18↓, TNF-α ↓ 3. lipid droplets and the NAFLD activity score↓, IPGTT and Fast Blood Glucose ↓ 4. the size and percentage of the lipid droplets↓	1*. Bifidobacterium*, *Parvibacter* ↑, 2. *Lachnoclostridium*, *Erysipelatoclostridium* ↓,	the pathways of primary and secondary BA biosynthesis were affected	(Chen et al., 2024a)
Huaganjian decoction	0.62, 1.24, 2.48 mg/kg/day by gavage for 4 weeks	HFD-diet SD male rats	1. body weight ↓, liver index↓, 2. serum ALT, AST, TC, TG, and LDL-C↓, HDL-c ↑ 3. hepatic lipid accumulation and TC, TG ↓	1. alpha diversity ↑, F/B ratio ↓, 2. *Lactobacillus* and *Allobaculum* ↓, *Bacteroidetes* ↑, *Proteobacteria* and *Tenericutes* ↑ 3. *Lactobacillu*s ↑, *Allobaculum* and *Paraprevotella* ↑, *Ruminococcus* ↓, *Blautia* ↓, *Oscillospira* ↓, *Bacteroides* ↓, *Clostridium* ↓, *Akkermansia* ↓, *Dorea* ↓, *Corynebacterium* ↓	1. total fecal BAs ↓, primary/secondary BAs ratio ↓, unconjugated/conjugated BAs ratio ↑ 2. alloLCA ↑, LCA ↑, isoLCA ↑, 7-k-LCA ↓, UCA ↓, TDCA ↓, GHDCA ↓	(Dong et al., 2025)

↑ indicates an increase or elevation, while ↓ indicates a decrease or reduction.

Emerging studies suggest the potential benefits of specific probiotic strains in NAFLD, such as *Bifidobacteria*, *Lactobacillus (*Yang et al., 2024*)*, *Faecalibacterium prausnitzii* (Martín et al., 2023), *Akkermansia muciniphila* (Cani et al., 2022), and *Clostridia* spp (Stoeva et al., 2021). For example, Xu et al. demonstrated that oral supplementation with *Bacteroides uniformis* and *Bifidobacterium bifidum* ameliorates hepatic pathological and metabolic disorders (Xu et al., 2024). Supplementation with *Lactobacillus plantarum* reduces the severity of NAFLD in HFD-induced mice (Zhao et al., 2020), while *Lactobacillus rhamnosus* GG supplementation activates the FXR-FGF15 signaling pathway to enhance BA excretion and prevent fibrosis (Liu et al., 2020). Furthermore, *Christensenella minuta* supplementation increases intestinal FXR-antagonizing acyl-BAs, counteracting HFD-induced metabolic dysfunction (Liu et al., 2024a).

## 6 Current limitations and translational challenges

Despite substantial progress in characterizing gut microbiota alterations in NAFLD, the reproducibility and translational value of these findings remain limited. This is due to pronounced inter−study heterogeneity. Discrepant observations, such as inconsistent changes in the *Firmicutes*/*Bacteroidetes* ratio, should not be interpreted simply as contradictory results. Instead, they reflect the multilayered biological and methodological complexity of the disease. At the biological level, age, sex, ethnicity, geographic region, host genetics, enterotype, obesity, insulin resistance, diabetes status, and disease stage may all shape gut microbial composition and functions (Sheng et al., 2024; Kabir et al., 2025; Kc and Paudel, 2025). At the environmental and clinical levels, dietary composition, physical activity, smoking, alcohol intake, antibiotics, antidiabetic drugs, lipid−lowering agents, and bariatric−surgery history may further modify microbial signatures (Zhang et al., 2024; Guo et al., 2025; Gupta and Gaur, 2025). In parallel, methodological differences in stool collection, storage conditions, DNA extraction, sequencing platforms, bioinformatic pipelines, sample size, and diagnostic criteria for NAFLD subphenotypes can substantially influence reported effect sizes (Panek et al., 2018; Gulyás et al., 2024; Guo et al., 2025). Reliance on broad taxonomic markers alone, especially phylum−level indices like the *Firmicutes*/*Bacteroidetes* ratio, is unlikely to generate robust biomarkers. Future studies should adopt standardized, multicenter, longitudinal cohorts with detailed dietary, pharmacological, and metabolic phenotyping, and should integrate shotgun metagenomics with metabolomics, transcriptomics, and BA profiling to identify reproducible functional signatures and clarify causal relationships within the gut–liver axis.

A similar limitation applies to preclinical modelling. No currently available murine model-whether diet−induced, genetically engineered, chemically induced, or based on combined insults-can simultaneously reproduce the full metabolic, histological, inflammatory, fibrotic, and oncogenic spectrum of human NAFLD/NASH. Some models generate steatohepatitis and fibrosis that resemble human liver histology but lack the metabolic background of obesity and insulin resistance, whereas others mimic metabolic dysfunction more faithfully but fail to recapitulate the complete sequence from steatosis to NASH, advanced fibrosis, cirrhosis, and hepatocellular carcinoma (Bhopale and Srinivasan, 2026). The methionine− and choline−deficient diet model is a representative example: it rapidly induces steatohepatitis and fibrosis, but is accompanied by weight loss and improved insulin sensitivity in mice—in contrast to the obesity and insulin resistance commonly observed in human NAFLD (Stephenson et al., 2018). Consequently, discordant findings across animal studies should not be regarded merely as experimental inconsistency, but as a consequence of model−specific applicability boundaries. Rather than seeking a single ideal model, future studies should select models according to the specific biological question being addressed, explicitly define the translational scope of each model, and standardize phenotypic assessment protocols, including metabolic parameters, histological scoring, inflammatory markers, fibrosis staging, and BA profiles. Such a strategy would improve cross−study comparability and enhance the reliability of preclinical evidence for therapeutic development.

Methodological variability in BA analysis represents another important but often underestimated source of inconsistency in NAFLD research. Peripheral BA concentrations and profiles are highly sensitive to preanalytical and analytical conditions. Fasting and post−prandial states can generate markedly different BA signatures, while the choice of serum or plasma, anticoagulant type, sample handling, storage temperature, freeze–thaw cycles and hemolysis status may further affect measured concentrations. Analytical procedures also introduce variability. Protein precipitation solvents such as methanol and acetonitrile may differentially influence the recovery and detectability of specific BA species-especially low−abundance conjugated, sulfated or glucuronidated BA-depending on the biological matrix and LC–MS/MS protocol. Therefore, differences in reported BA profiles across studies may reflect not only biological variation but also method−induced bias. To improve reproducibility, future investigations should standardize sampling time, fasting duration, blood−matrix selection, processing procedures, storage conditions, internal standard selection, calibration ranges, limits of detection and quantification, recovery, matrix effects and inter−batch coefficients of variation (Joseph et al., 2025).

These methodological and model−related limitations are particularly relevant when interpreting FXR−targeted therapies. FXR is not a uniformly beneficial or deleterious target; rather, its metabolic effects are tissue−specific, ligand−dependent and disease−stage−dependent. Hepatic FXR activation suppresses BA synthesis, lipogenesis and inflammatory signaling, thereby protecting against steatosis and hepatocellular injury (Armstrong and Guo, 2017; Tanaka et al., 2017). In contrast, intestinal FXR activation exerts more complex effects. Through the FGF15/19−mediated feedback pathway, intestinal FXR limits hepatic BA synthesis and modulates hepatic glycogen and fatty−acid metabolism (Bozadjieva et al., 2018). However, intestinal FXR activation may also suppress glucose−stimulated GLP−1 secretion from enteroendocrine L cells, whereas TGR5 activation promotes GLP−1 release (Trabelsi et al., 2015; Radun and Trauner, 2021). Conversely, in mouse models, intestinal FXR antagonism by BA or intestine−restricted FXR inhibitors has been associated with reduced circulating ceramides, decreased hepatic gluconeogenesis, improved insulin sensitivity, enhanced GLP−1 secretion and attenuation of hepatic steatosis (Wu et al., 2021b). Nevertheless, these findings should be interpreted cautiously because murine and human BA pools differ substantially, particularly concerning muricholic−acid species. Thus, mechanisms involving tauro−β−muricholic acid or related murine BA–FXR interactions may not be directly extrapolatable to humans.

This tissue−specific complexity helps explain why clinical translation of FXR agonists in NAFLD has been challenging. Global FXR activation may produce desirable antifibrotic and metabolic effects in some contexts, but these benefits must be balanced against adverse events such as pruritus, dyslipidemia, potential glycemic deterioration, gallstone−related events and cholestatic safety concerns (Tian et al., 2022). Clinical experience with FXR agonist monotherapy, particularly OCA, suggests that improvement in fibrosis may be achievable, but histological NASH resolution and an acceptable long−term benefit–risk profile have not been consistently demonstrated. Moreover, the therapeutic landscape of NASH is rapidly evolving with the approval of non−FXR−based therapies, including thyroid hormone receptor−β agonists and GLP−1 receptor−agonist approaches, further raising the bar for FXR−targeted agents. Therefore, the future value of FXR modulation is unlikely to depend on nonselective systemic FXR activation alone. More promising strategies may include tissue−selective FXR modulation, intestine−restricted FXR antagonism or agonism according to metabolic context, dual− or multi−target agents such as FXR/TGR5 modulators and combination regimens designed to preserve antifibrotic efficacy while mitigating dyslipidemia, pruritus and cholestatic risk.

Collectively, these challenges indicate that NAFLD should not be approached as a single homogeneous disease entity, nor should FXR be regarded as a uniformly protective target. Variability in host phenotype, gut microbial ecology, BA metabolism, experimental models, analytical methods and tissue−specific receptor signaling all contribute to divergent findings across studies. Future research should therefore prioritize standardized human cohorts, mechanistically informed animal−model selection, harmonized BA analytical protocols and integrative multi−omics approaches. In parallel, clinical development of FXR−targeted therapies should move beyond global agonism toward precision modulation of hepatic and intestinal FXR pathways, with careful attention to disease stage, metabolic background, BA pool composition and long−term safety. Such a framework may improve the reproducibility of mechanistic studies and facilitate the rational translation of gut–liver axis biology into effective therapies for NAFLD.

## 7 Conclusions and future perspectives

This review integrates current evidence on the gut microbiota–BA–FXR axis in the pathogenesis and therapeutic modulation of NAFLD. Rather than viewing BAs only as digestive molecules or treating microbial dysbiosis as a secondary consequence of metabolic disease, this axis provides a mechanistic framework linking intestinal ecology, BA remodeling, nuclear receptor signaling, and hepatic metabolic injury. Altered BA composition, particularly changes in the balance between 12-hydroxylated and non-12-hydroxylated species, appears closely associated with insulin resistance, hepatic lipogenesis, inflammation, and hepatocellular stress. FXR acts as a central regulatory node within this network, but its effects differ across tissues and metabolic contexts. Hepatic FXR activation generally restrains BA synthesis, lipid accumulation, and inflammatory signaling, whereas intestinal FXR signaling can produce more divergent metabolic outcomes. These findings support a more nuanced view of the gut microbiota–BA–FXR axis: it is not a linear pathway, but a dynamic regulatory network that shapes disease progression and offers several points for therapeutic intervention.

Future work should shift from descriptive association to mechanistic validation and clinically actionable stratification. Large, longitudinal, and well-phenotyped cohorts with standardized stool, serum, plasma, and BA analytical protocols are needed to define reproducible microbial and BA signatures across NAFLD stages. Mechanistic studies should use gnotobiotic models, humanized microbiota systems, organoids, and targeted genetic approaches to identify the bacterial enzymes, BA species, and FXR-dependent pathways that directly influence steatosis, inflammation, and fibrosis. Therapeutic development should move beyond nonselective systemic FXR activation toward tissue-selective FXR modulators, intestine-restricted strategies, engineered probiotics or postbiotics, and rational combinations with lifestyle intervention and emerging metabolic therapies. Equally important, future trials must assess long-term safety, interindividual variability, and disease-stage specificity. By integrating multi-omics data with clinical phenotyping and computational modeling, the field may translate gut–liver axis biology into more precise biomarkers and individualized treatment strategies for NAFLD.
